# The Matron's Department

**Published:** 1906-01-13

**Authors:** 


### HOSPITAL ADMINISTRATION.

### CONSTRUCTION AND ECONOMICS.

### THE MATRON'S DEPARTMENT.

#### XVI.—THE MATRON'S DUTY TOWARDS HERSELF.

All administrative work demands the power to make use of imperfect instruments, but no one will make a good administrator who has not discovered that self is the most imperfect of them all. To fail again and again and again, and each time to search diligently, not for some scapegoat on whom to lay the blame, but for the latent blunder which led on to failure, this is the only road to successful rule. Who cannot look back on early experiences and see in the light of later wisdom that nine times out of ten when things went wrong it was because of some precaution omitted, some detail dropped out of memory, some subordinate too rashly trusted? To be for ever providing against contingencies, and learning from each reverse to take ever greater precautions in the future, is the task of all who aspire to the tranquil self-confidence of the capable ruler. The beautiful repose of manner characteristic of a woman sure of herself is not attained without conflict. It results from a trained habit of mind, and is within the reach of all who will learn the secret of training self through the medium of training others.

Meantime it saves a great deal of heart-burning and chafing against the established order of things if the matron lay to heart from the first moment of her appointment the great truth that, to use the words of an American essayist, "There's a deal of human nature in man." Every one of her subordinates, every one of her colleagues has a seamy side. Of nearly every one in the house she will be tempted to say at one time or another, "Things would be so much easier if so and so were different." And yet it is in this imperfect microcosm that she, imperfect herself, has been appointed to work, and it is from just such a collection of imperfect instruments that the highest service to God and man is daily all round us being performed. In the eternal conflict against evil, through which all that is worth doing has to be accomplished, the first step to victory is to discern the foe. Hence first among the duties of the matron stands the duty of dealing wisely with defects in character, defects in knowledge, defects in manner, among those with whom she is to live.

And because little things are always far harder to bear philosophically than big ones, it is defects in manner which will irk her most. Racial differences flourishing all unconsciously as between one part of the country and another; differences of tradition developed by training in various institutions; differences in education and early upbringing—all these have to be taken into account. And again, there are many curious and contradictory strands of convention even in this country in the twentieth century whereby one person's code of politeness stands to another as a cause of offence. Zeal for a common object does much to obviate strain, but perhaps a sense of the humour of things does even more towards lifting the mind above superficial annoyances—the humour, that is to say, which seizes on the kinship of minds and laughs with, not at, another.

If defects in manner can be ignored, defects in knowledge and habit must be repaired, and it has been the object of the foregoing pages to show through what means the matron may help to shape uninstructed members of her household to serve a common end. Never to feel personally aggrieved by imperfection, and never to grow impatient in supervision and correction, this is the unending task in which every head of an institution must brace herself to spend her days. But of this enough has been said. It is sufficient reward as time goes on to watch raw material develop into instruments of the highest value.

And as regards faults of character, untruthfulness, backbiting, vanity, want of loyalty, ill-temper, sloth, self-seeking, is it not the lot of all to be compelled to work day by day with men and women displaying such characteristics? The matron will be unusually fortunate if there are not among the members of the household some whom her judgment cannot approve. Perhaps one of her most painful duties will be that of finally deciding against such as are unamenable to discipline and adverse to the interests of the institution. It needs all the best powers of the ruler to prevent patience from degenerating into weakness in cases where dismissal is advisable, and to choose the right moment and motive for action, and there are hardly any circumstances under which mistakes are more frequent and more deplorable. Experience is a safe guide, but for the comfort of the inexperienced be it said that the habit of justice is even safer, and justice is much more a matter of taking pains than is generally supposed. To get at all the facts first hand, to give her whose fate is under consideration opportunity for stating her own case, to sum up carefully for and against, to take that diligent care which is never superfluous, to exclude all personal considerations, and then to act decidedly and kindly, all this means trouble, but it is the only road by which to acquire the judicial attitude. It is a very common error with kindhearted women to condone offences long after it has become evident that the offender is neither doing nor getting any good. It is often far kinder in reality to terminate the situation, recognising that those who fail under one set of circumstances may do very well indeed when conditions are changed, and that the shock of acknowledged failure may be the first step towards reform.

But there will be cases in which those who are the objects of just suspicion and dislike do not lie under the matron's jurisdiction, yet must be borne with in daily intercourse, perhaps obeyed. In such cases her duty is clear. She cannot lower her own tone in compliance with sentiments she disapproves, but neither is she called on to step outside her own sphere to argue or protest. If her own light is steady, sooner or later it will absorb the shadows. The influence of a just and sincere woman at the head of the household will make itself felt even to the remotest corner; nor is any malign example able to withstand it. In quietness and in confidence shall be her strength.

We have dealt thus long on the subject of other people's faults because it is those which in innumerable cases pave the way to disappointment and failure. The wise ruler must be strong enough to keep her work unmarred by others. But though it be her duty thus to open her eyes to evil, it is her happy privilege, working as she must do in closest association with others, to penetrate to an intimate understanding of their higher nature. There is perhaps no other class of work which develops, to the same extent as nursing, qualities of the heart; and the mutual spirit of confidence and dependence between the matron and her sisters when each has learned to trust the other renders this one of the happiest relations life has to offer. It is matter of common observation that the women who govern well are the women who are quick to see the good points in others and slow to condemn. Any shallow critic can be relied on to detect faults. But good judgment is needed "to pick out treasures from an earthen pot." It is certainly one of the many interesting features in the training of probationers that it tends to bring to light qualities previously unguessed at by themselves or by their friends. And if the matron is to get the best out of her material she must have faith in these dormant virtues and learn to awake them. So true is it that belief in their existence suffices often to warm them into life.

And when all has been said of the matron's life with others and her duty towards herself as displayed in her intercourse with her little world, what can be said of that which is more to her than all the rest, the inner life which is lived all day long in the secret places of her soul? How much must be surrendered to the exigencies of the work? It is inevitable that much must be sacrificed by the head of a large household. Favourite pursuits will perforce dwindle to occasional recreations. Friends will come to recognise that even friendship must be subordinated to higher claims. Amusements of at all an exciting or engrossing character will gradually be foregone. And the routine life of the hospital, largely made up as it is of emergencies, will come to bound the horizon to the exclusion of vivid outside interests. We believe this to be inevitable, but not for that reason need the nature of a busy woman be warped and her outlook on life narrowed. It is the spirit in which daily duties are encountered, not the occasional excursions from them, which has power to broaden and deepen the whole nature. Such excursions from the narrow things of home, wholesome and, to some natures, even necessary, if the equilibrium is to be maintained, tend to become more rarely tasted as the sense of responsibility grows with the consciousness of administrative ability. But if the inner life is to remain serene, unmarred by strife of tongues, some period every day, some true holiday at least once a year, must be secured wherein the soul may be uplifted above the smoke and stir of this dim spot. It matters little by what method tranquillity is wooed: to each nature its own mode of solace. But only through such reviving spells of self-realisation can tranquillity develop from the joy of occasional rare moments to the habit outside influences shall have no power to destroy. This is that inward vision, source of all real power over men and things, wherein all happenings assume their true proportion and the soul reaches forward towards perfection.

